# Communicative and social competence in the medical curriculum of the Medical University of Innsbruck: learning objectives, content, and teaching methods

**DOI:** 10.3205/zma001455

**Published:** 2021-03-15

**Authors:** Silvia Exenberger, Martin Kumnig, Alexandra Huber, Wolfgang M. Prodinger, Heidi Siller, Elisabeth Medicus, Erich Brenner, Gerhard Schüßler, Stefan Höfer

**Affiliations:** 1 Medical University Innsbruck, Department of Medical Psychology, Innsbruck, Austria; 2 Medical University Innsbruck, Department of Hygiene and Medical Microbiology, Innsbruck, Austria; 3 Medical University Innsbruck, Gender Medicine Unit, Innsbruck, Austria; 4 Medical University Innsbruck, Innsbruck, Austria; 5 Medical University Innsbruck, Institute for Clinical and Functional Anatomy, Innsbruck, Austria

**Keywords:** communicative competencies, medical studies, communication skills, assessment, ethics, gender identity, palliative care

## Abstract

**Aim: **The Austrian Competence Level Catalogue for Medical Skills clearly states the importance of teaching communicative and social competence in the different subject areas of undergraduate medical and dental education. This paper aims to present an overview of the academic courses at the Medical University of Innsbruck that explicitly address the promotion of communication and social skills in medical students.

**Method: **This paper focuses on educators’ descriptions of how communicating with patients is taught. The Medical University’s longitudinal curriculum on medical interviewing is presented in detail. The courses on ethical principles in the dissection course, palliative medicine, and gender medicine are also outlined as examples. In addition, lecturers (n=536) participated in an online survey to determine the teaching and testing content regarding patient communication and to measure the value attached to the associated teaching and learning methods.

**Results:** The examples given by educators to illustrate learning objectives, educational content, and the teaching methods used to impart communicative and social competence provide an overview of the courses which focus on this topic or intentionally address it during the course. The results of the online survey offer a broad overview of the awareness of the topic at the university. Different testing formats are used to assess the skills being taught.

**Conclusion:** Familiarity with the various teaching methods used in the different courses is important for developing communicative and social competence in medical education. Active networking is necessary to anchor communicative and social competency as a major thread throughout an entire medical curriculum.

## Zusammenfassung

**Zielsetzung: **Der Österreichische Kompetenzlevelkatalog für ärztliche Fertigkeiten benennt klar die Bedeutung des Trainings kommunikativer und sozialer Kompetenzen für die verschiedenen Lehrbereiche der Grundstudien Human- und Zahnmedizin. Dieser Beitrag hat zum Ziel, einen Überblick über Lehrveranstaltungen an der Medizinischen Universität Innsbruck zu geben, die explizit die Förderung von kommunikativen und sozialen Kompetenzen bei Medizinstudierenden zum Thema haben.

**Methodik: **Im Fokus dieses Beitrages stehen Beschreibungen durch die entsprechenden Dozierenden wie Kommunikation mit Patient*innen gelehrt wird. Ausführlich wird das Längsschnittcurriculum Ärztliche Gesprächsführung dargestellt. Beispielhaft werden die Lehrveranstaltungen der teils longitudinalen Curricula ethische Grundlagen im Sezierkurs, Palliativmedizin und Gender Medizin skizziert. Außerdem nahmen Dozierende (N=536) an einer Onlinebefragung teil, um Lehr- und Prüfungsinhalte zur Kommunikation mit Patient*innen abzubilden sowie den Stellenwert assoziierter Lehr- und Lernmethoden zu erfassen.

**Ergebnisse:** Die beispielhafte Beschreibung der Lehrenden von Lernzielen, Lehrinhalten und der verwendeten Didaktik für die Vermittlung kommunikativer und sozialer Kompetenzen gewährt einen Überblick über jene Lehrveranstaltungen, die einen Schwerpunkt auf dieses Themen legen bzw. das Thema in der Lehrveranstaltung bewusst ansprechen. Die Ergebnisse der Onlinebefragung geben einen groben Überblick zur Bewusstheit des Themas an der Universität. Die gelehrten Kompetenzen sind Bestandteile unterschiedlicher Prüfungsformate.

**Schlussfolgerung: **Für die Weiterentwicklung kommunikativer und sozialer Kompetenzen in der Ausbildung von Medizinstudierenden ist es wichtig, die Lehrkonzepte der jeweiligen Lehrveranstaltungen untereinander zu kennen. Aktive Vernetzungsarbeit ist notwendig, um kommunikative und soziale Kompetenzen als roten Faden durchgängig in einem Medizincurriculum zu verankern.

## 1. Introduction

In the course of practicing medicine, physicians constantly see that professional communication with patients is essential. It is, for instance, through communication that the psychosocial basis is created for a patient’s future compliance with doctor’s orders and enhancing patients control belief [1]. The consequences of successful versus unsuccessful doctor-patient communication has been repeatedly documented ([2]; for an overview: [3]). Di Blasi and Kleijnen (2003) have been able to show a healing effect of doctor-patient interactions and view them as a powerful trigger for self-healing processes [4]. Even in an era of highly technological medicine, doctor-patient consultations represent the most commonly used medical intervention. Since all recommendations for professional medical education assume that communication between a physician and patient can be learned, the teaching of communicative competence is a component of almost all reformed curricula [5], [6]. The Austrian Competency Level Catalogue for Medical Skills [7] – which contains the basic common learning objectives required by the Austrian medical schools – assigns a clear place for communication and social skills, as is the case in other German-speaking countries [8].

This paper addresses the question of how the basic requirements have been implemented at the Medical University of Innsbruck. The aim is to give an overview of the courses that focus explicitly on promoting communicative and social competence in medical students. It can also serve as motivation for other educators to directly teach communication with patients in different courses.

## 2. Method

Two methodical steps were selected to answer the question and fulfill the aims:

1. In the first step, an online survey of lecturers was conducted to collect information about communicative and social competences in academic teaching at the Medical University of Innsbruck. This was done to gain an overview of the course offerings regarding communication and social skills (see attachment 1 ). The online-questionnaire consisted of 14 questions on current teaching and testing content pertaining to communication with patients and the value placed on this topic at the university, as well as its importance from the perspective of the educators. In January 2020 the survey was sent by the office of the Vice Dean to all medical and dental educators (n=536) and carried out using LimeSurvey 3.17.2 [9].
2. In the second step, the sequence of courses dedicated to holding medical interviews was described in detail. This involves the only course that explicitly and exclusively focuses on doctor-patient communication. In addition, the lecturers were invited to briefly describe their courses if they addressed the topic of communicating with patients. Three educators (palliative care, ethics in the dissection course, gender medicine) accepted this invitation.

## 3. Results

First, the results of the online survey, which provide a broad overview of the issue, are presented. Following this are the detailed descriptions of the longitudinal curriculum on doctor-patient communication and the three courses that cover this topic.

### 3.1. Online survey

Out of a total of 536 lecturers, 256 participated (47.8%). For approximately half of the participants (n=106; 41.4%), the topic of communicating with patients is intentionally covered in their teaching. Of these lecturers, the majority (total n=97; 91.5%) hold two to four corresponding courses (modal value=3) per semester using different teaching formats (modules, practical courses, lectures, etc.), which are attended by five to 420 students. The number of students in each course depends on the type of course (practical course vs. lecture). The skills for communicating with patients are primarily taught in lectures (n=60) and discussions (n=59). In addition, patients are presented (n=39) and case vignettes (n=39) are used. Bedside teaching (n=29) and covering course topics in small groups (small-group work, n=22) were also frequently used as methods. Role-playing with fellow students (n=12) and simulated patients (n=9) were used much less often. Most rarely used was video analysis of consultations (n=7), in which the students themselves assumed the role of the physician or recorded interviews were discussed in a group setting for the purpose of learning.

The online survey of the different teaching formats showed that written tests (n=41) and oral exams (n=37) were used most often; a combination of the two (n=3) was used most seldom. A practical assessment, for instance an Objective Structured Clinical Examination (OSCE), was mentioned 22 times. Although there is consensus among the survey respondents that the teaching of communicative and social competence is of great importance, a large percentage of the lecturers (total n=83) had very little or no precise knowledge about the relevant required (n=66) and elective courses (n=72).

#### 3.2. Description of curricular courses at the Medical University of Innsbruck

The curricula for undergraduate study in medicine and dentistry focus early on the development of psychosocial competence, such as learning about the health occupations and the ethical principles of medical practice. Practical training and specific training in conducting medical interviews lead – together with the acquisition of other general skills (e.g., drawing blood) –to attaining the clinical clerkship competence level at the end of the fourth semester. It is at this time that students undergo assessment by taking the Clerkship OSCE administered in compliance with the Austrian Competence Level Catalogue [7].

Basically, communication takes place in the different subject areas taught in medicine and dentistry. On the one hand, this involves courses that implicitly teach communication (e.g., surgical anamnesis); whereas other courses explicitly identify communication as learning content (e.g., the longitudinal curriculum in medical interviewing). In the following we describe the required medical interviewing curriculum in detail and three other courses as examples.

##### 3.2.1. Longitudinal curriculum on holding medical interviews

The currently required courses on conducting medical interviews are described in the undergraduate medical curriculum (academic year 2018/2019 [10]). The curriculum’s courses, which are sequenced over several semesters, aim to impart skills and abilities pertaining to medical interviews. An introduction to theory is provided in the lecture course on how to deal with a sick person; the longitudinal curriculum picks up again in the third semester and is covered each semester in small groups of six to 12 students until the end of the seventh semester (see table 1 (Tab. 1)).

The theoretical basis underpinning doctor-patient communication is covered in the lecture courses that are held in the first and third semesters. In the practical courses the students are guided by lecturers who have received training on how to teach communication techniques. In addition, patient interviews are monitored by trained and clinically supervised tutors.

A core component of the practical courses is the opportunity to practice with simulated patients (SP). Standard situations, such as taking case histories, advising and counseling patients, and the delivery of bad news, are practiced under realistic conditions. Critical moments in a conversation (teachable moments) are used to help students find opportunities to improve.

The competence gained by students is measured on an OSCE. This training contributes significantly to patient safety since, prior to interacting with real patients, all of the students have practiced the necessary steps in a simulated environment. The curricular courses teach the basic elements and skills for successful patient-centered communication (see table 2 (Tab. 2)). In the first part of the curriculum, students practice taking biopsychosocial histories, in part with real patients. The students prepare a written case history that is then graded. For the students this is the first structured interaction with patients.

One special educational aspect of the practical courses in the longitudinal curriculum is feedback training. In all of the interactive exercises on communication, students are required to share their perceptions and experiences of others (give feedback) and to listen to how they themselves are seen by others (receive feedback). In role-play exercises students assume the role of physician and reflect on the positive and negative elements of their behavior. So that students learn the rules of giving and receiving feedback, an online feedback course was created by the Department of Medical Psychology, which all students are required to successfully complete as part of the first practical course in medical interviewing. The feedback rules are introduced and demonstrated using videos as examples. At the end, the students formulate their own feedback on a specific video sequence and this is included in the grade for the practical medical interviewing course. Care is taken to make good use of the feedback rules in all of the subsequent practical courses on medical interviewing.

There is a systematic evaluation of learning outcomes since a large portion of the courses in the medical interviewing curriculum cover communication as a topic, train social skills in physicians-to-be, and are exam-relevant required courses within the curriculum. Students’ active participation in the individual courses is evaluated and they receive structured feedback on their performance from lecturers (e.g., as part of video analyses). While the video analyses serve as a teaching strategy in the courses in semesters 3 through 6, the video analyses during the final course in the medical interviewing curriculum are also used to evaluate learning gains in addition to student participation.

An interdisciplinary OSCE is administered at the end of the fourth semester to assess students’ readiness for the clinical clerkship. This assessment encompasses core medical skills such as taking a biopsychosocial anamnesis with the use of SP. The interaction with the SP is graded based on standardized checklists and counts toward the total station score. To ensure the objectivity and reliability of the test situation, the SPs receive regular training on the current exam content.

In the tenth semester and prior to the final year of study (clinical practical year), which is spent at different teaching hospitals, students take an interdisciplinary OSCE covering 11 clinical subjects at eight stations. On the OSCE stations with SP (approximately 70% of the stations) the interactions with the SP are graded in the same way using a checklist and count toward the total score for each station.

In medical and dental education there are ongoing measures to ensure the quality of the teaching in the medical interviewing curriculum. From 2010 to 2012 a teaching project took place within the scope of the Clinical Skills Lab to give students the opportunity to acquire interviewing skills with SP. The project enabled targeted post-licensure education (e.g., European teaching and testing standards) for educators and the development of new teaching techniques (e.g., feedback training) and materials. At the time, these measures were evaluated in terms of the influence of the changes to the curriculum and teaching methods over time on students’ experience of communicative and social competence. After implementing the opportunity to practice with SPs, the students at that time reported on average a significantly higher level of confidence regarding their communication skills than before. They benefited primarily in the area of personal development, preparation for contact with patients, and new interviewing techniques.

##### 3.2.2. Palliative medicine

When caring for patients with a terminal illness, communication with them and their relatives is vital [11]. Specific communication topics in palliative care include conveying the prognosis, defining the goal of therapy in ambiguous situations in tandem with the patient, anticipating crises (advance care planning), and conversations about how the final phase of life up until death should be handled [12], [13].

Palliative medicine is a required subject in medical education (8^th^ semester). About 15-20% of the subject content refers to these specific communication topics (in compliance with the recommendations of the European Association of Palliative Care, EAPC [14]), which are taught as theory and using case vignettes as examples. Communication models are also used [15], [16].

This knowledge is covered in more depth in the elective seminar on palliative medicine. This includes exercises in active listening, contact with family members and, if possible, interaction with patients to accomplish a clear communicative task depending on the given situation (e.g., wish to die), and a final discussion and analysis of these patient interactions in the seminar. Strong, sometimes conflicting emotions, as they occur in connection with the experience of loss in patients and their relatives, require a social and reflective competence regarding one’s own attitude toward death and dying and the role of physicians in providing care at the end of life.

##### 3.2.3. Ethical principles in the dissection course

The topographic dissection course that medical students are required to take in the third semester (9.5 hours per week per semester) provides an opportunity–based on its intensity and small group setting–to integrate communicative and social competence. Already in 2003 the institute staff used the Medical University’s list of qualifications (as a set of general learning objectives) to identify intermediate teaching objectives and defined several in the area of communicative competence [17]:

1. observe and justify the ways of behaving toward the corpse,
2. express oneself precisely and understandably in both spoken and written language using clinical and scientific terminology, and
3. appropriately and precisely document activities.

Since these skills cannot be assessed via the usual oral and/or spotter tests, individual weekly structured observations and a small-group portfolio assignment were introduced [18]. Similar to the implementation at other medical schools, a parallel course – Ethics in the Dissection Course – was created. The dissection course was revised in 2019 and is now based on the CanMEDs 2015 Framework (Canadian Medical Education Directives for Specialists). In doing this, the ability to document and report patient interactions in writing and electronically to optimize clinical reasoning, patient safety, confidentiality and data protection were taken from the competencies of the “communicator” and applied to the context of the dissection course [19]. Students practice these skills on all of the assignments in team-based learning, meaning that all of the texts in the small-group portfolio must be finished on time and well written. The private sphere of the donor should always be respected, something that will be of utmost importance later when providing medical care to patients [20].

##### 3.2.4. Gender medicine

Gender Medicine (GM) focuses on the commonalities and differences between sex (biological sex) and gender (socio-cultural concept of sex) regarding “normal” function, disease, diagnostics, and treatment of diseases [21]. In recent years GM has been expanded to include additional aspects of diversity (e.g., origin). These sex-, gender- and diversity-specific characteristics are part of medical education [22] and research [23], [24], as well as the doctor-patient relationship. Taking sex, gender and diversity characteristics into consideration during patient consultations is recommended [25]. GM is a longitudinally implemented subject in all medical curricula. This is done to sustainably anchor knowledge of relevant definitions and sex-, gender-, and diversity-specific diagnostics and therapy (e.g., gender bias in diagnostics), awareness and reflective thinking during interactions with patients (e.g., heteronormative assumptions), and sensitivity for these characteristics into the research. In addition to the lecture to impart knowledge and awareness of sex-, gender-, and diversity-specific characteristics in disease and therapy in the third and ninth semesters of medical study, and in the first and third semesters of the bachelor’s program in Molecular Medicine, seminars cover some areas in more depth. Elective seminars cover topics on sexuality or diversity in reference to resilience, migration, and cultural competence in order to elucidate gender- and diversity-specific aspects that manifest in attempts to seek help, in hierarchies, and in privilege. The required seminar focuses on gender-specific aspects of interpersonal violence in that the handling of gender- and diversity-specific aspects is also discussed in practical terms and, thus, the topics of cultural and social competence are raised. In the PhD seminars students apply their knowledge of sex-, gender- or diversity-specific characteristics to their area of research.

## 4. Discussion

### 4.1. Organization and structural anchoring

It can be seen that no further course explicitly centered on communicative and social competence is offered later in the curricula for undergraduate medical and dental education. There are different reasons for this such as, for instance, the fact that this kind of teaching requires intensive staff resources and increased lecturer training so that they can adequately teach the technology and professional attitudes [26]. The ongoing continuation of teaching with an explicit focus on communicative and social competence over the entire course of study would, however, be necessary since it is only through critical and repeated practice that a medical approach with “heart and hand” can be achieved to counteract structural deficits in the learning of communication skills [27].

#### 4.2. Quality assurance and evaluation

Ongoing internal measures to ensure quality include continuing education and training sessions for lecturers of the medical interviewing curriculum (e.g., Basel Consensus Statement, [6]) and feedback training to develop roles for SP according to the Heidelberg model [28]. In addition to general theoretical training, new lecturers and peer tutors receive specific practical lecturer training on the courses or tutorials they will later give on their own. Doing this ensures that all of the course teaching will align as closely as possible with the educational concepts embraced by the medical interviewing curriculum.

#### 4.3. Has the communication culture changed?

Up until 2002 the medical and dental curricula contained no standardized training in interviewing and patient examinations. Since then, the importance of teaching communication has increased as a result of introducing the medical interviewing curriculum and expanded the integration of teaching communicative and social competence in other medical subjects. A total of 60% of the respondents to the online survey conducted in January 2020 at the Medical University of Innsbruck on communicative and social competence stated that the curricular content addressing communication with patients was viewed as important or very important by the university. The teaching of communicative and social competence during medical study is important or very important to 99% of the respondents.

Basic interviewing skills, such as independently taking a medical history and the use of specific intervention techniques, are viewed by students as basic pre-requisites for future medical practice. The teaching methods applied here enable students to safely practice interacting with patients in challenging situations, something that could hardly be done in real clinical practice (e.g., delivering a cancer diagnosis). In feedback rounds students also receive clear information about their patient interviews so that they can reflect again on their skills and then work on them further in practice sessions.

Based on the results of the quality assurance measures and course evaluation it could be confirmed that not only student interest in conducting medical interviews with patients has increased, but student performance has also improved as a result of the systematic teaching.

The result of the online survey shows that lecturers know hardly anything at all about the required and elective courses covering communicative and social competence and makes it clear that there is a need for networking among lecturers regarding this topic. Even if a large percentage of the survey respondents claimed to have diverse educations (e.g., psychotherapy) and thus viewed themselves as having qualified skills in teaching medical communication techniques, only a minority of them took advantage of specific additional lecturer training programs on how to teach communication techniques in the context of medical education. We recommend more training programs for lecturers that focus on how to teach communicative and social competence so that they intentionally include it in their courses and explicitly invest themselves in the teaching of it.

Medical education and the practice of medicine will radically change—mainly as a result of digitalization. The value placed on effective communication between doctors and their patients will therefore increase for future physicians. As a consequence, university education must prioritize this aspect much more strongly in the future.

## Profiles

**Institution: **Medical University of Innsbruck, Innsbruck, Austria.

**Study program/occupational group: **Medicine, Dentistry, Molecular Medicine.

**Number of lecturers per year or semester: **Number of academic staff at the entire Medical University who are engaged to differing degrees in teaching: 1,100; supported by 200 student employees (tutors) in varying part-time capacities.

**Has a longitudinal communication curriculum been implemented?** Yes.

**In which semesters is communicative and social competence taught?** Semesters 1, 3, 4, 6 & 7 in the undergraduate medical degree and dental degree programs.

**Which teaching formats are used?** Lecture, group work, role play, discussion, work with simulated patients, video training, bedside teaching.

**In which semesters is communicative and social competence assessed? **Third-semester OSCE (dentistry); fourth-semester OSCE (medicine); tenth-semester OSCE (medicine); test-like character in semesters 3, 4 and 6 of the medical and dental degree programs, semester 7 in medicine.

**Which assessment formats are used? **Graded participation, video-recorded test interview, OSCE.

**Who is entrusted with development and implementation?** In terms of content, the University Clinic for Medical Psychology (development, implementation, ongoing development); in terms of structure, the Curricular Commission (for the overall curriculum) and the Vice Dean (for lecture coordination and organization).

## Current professional roles of the authors

- Mag.a Dr.in rer. nat. Silvia Exenberger: Department of Medical Psychology, Medical University, Innsbruck. Clinical and health psychologist
- Priv.-Doz. Mag. Dr. phil. Martin Kumnig: Department of Medical Psychology, Medical University Innsbruck, Clinical and health psychologist
- Mag.a Dr.in rer. nat. Alexandra Huber: Department of Medical Psychology, Medical University Innsbruck, Clinical and health psychologist
- ao. Univ.-Prof. Dr. med. univ. Wolfgang Prodinger, MME (Bern): Department of Hygiene and Medical Microbiology, Medical University Innsbruck, and Curricular Commission, Medical University Innsbruck
- Mag.a Dr.in rer. nat. Heidi Siller: Gender Medicine Unit, Medical University Innsbruck, Clinical and health psychologist
- Dr.in med. Elisabeth Medicus: General practitioner, Palliative medicine specialist, Lecturer at Medical University Innsbruck
- ao. Univ.-Prof. Dr. med. univ. Erich Brenner, MME (Bern): Institute for Clinical and Functional Anatomy, Medical University Innsbruck
- o. Univ. Prof. Dr. med. univ. Gerhard Schüßler: Head of the Department of Medical Psychology, Medical University Innsbruck
- Assoz.-Prof. Priv-Doz. Mag. Dr. rer. nat. Stefan Höfer: Department of Medical Psychology, Medical University Innsbruck, Clinical and health psychologist

## Competing interests

The authors declare that they have no competing interests. 

## Supplementary Material

Questionnaire

## Figures and Tables

**Table 1:**
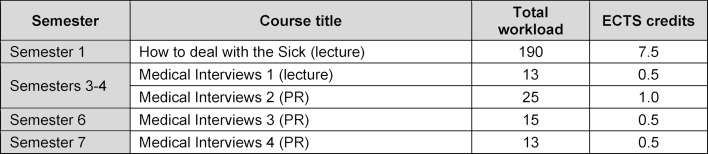
Study plan human medicine

**Table 2:**
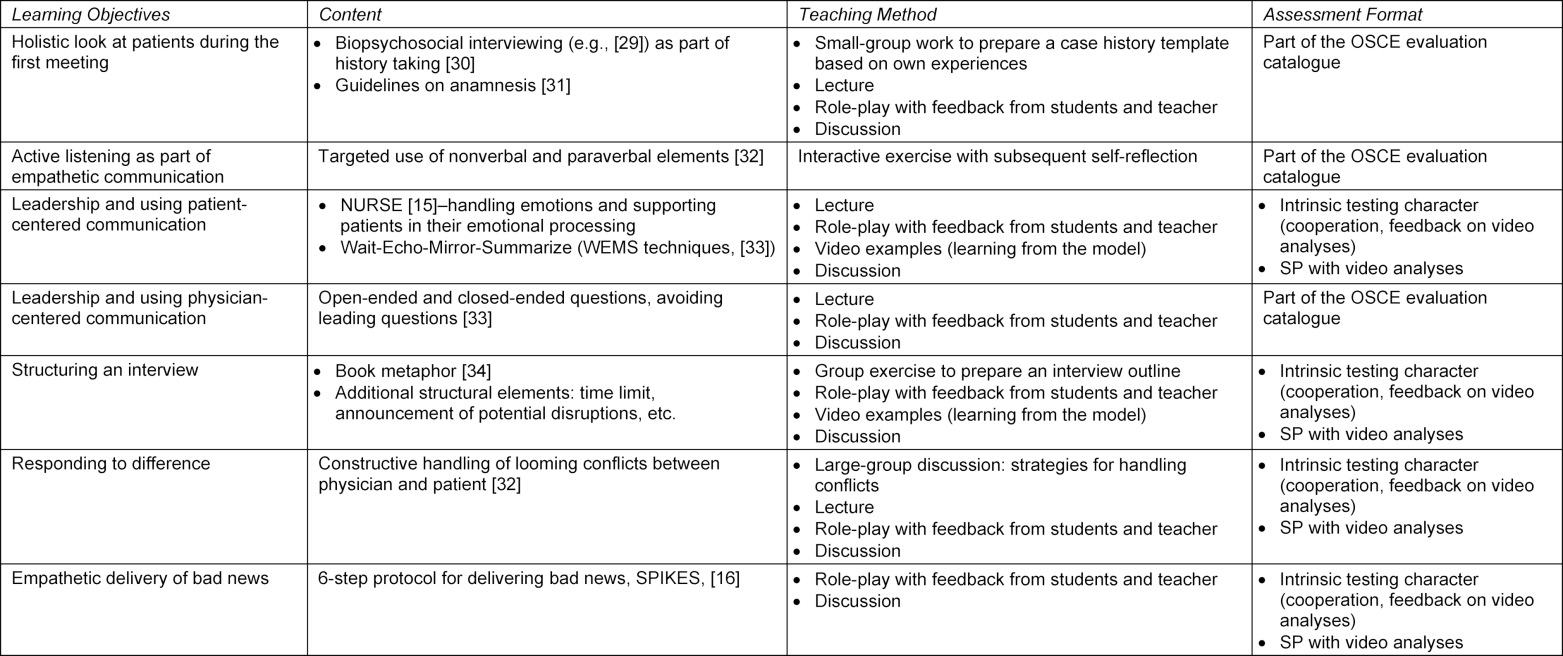
Training sequence of the learning events
