# Supplementary material for: Communicative and social competence in the medical curriculum of the Medical University of Innsbruck: learning objectives, content, and teaching methods
Source: GMS J Med Educ. 2021 Mar 15;38(3):Doc59. doi: 10.3205/zma001455 (PMC7994870; doi:10.3205/zma001455)
Supplement: Questionnaire [file JME-38-3-59-s-001.pdf]

Dear Lecturers,

We would like to present the Medical University's current teaching and testing content on communication with patients in a paper to be published in the Journal for Medical Education's special issue on Communicative and Social Competence. To do this we will need the help of *all* teachers.

We kindly request that you fill out this brief survey to the best of your knowledge.

Thank you very much!

Dr. Silvia Exenberger, Dr. Alexandra Huber, Dr. Stefan Höfer, Dr. Gerhard Schüßler, Dr. Wolfgang Prodinger, Dr. Peter Loidl

Part A: Communicative Competence

A1. Is "communicating with patients" a topic that you intentionally cover in the courses (modules, lectures, practical courses, seminars, etc.) that you teach yourself or in which you participate (e.g. as a guest teacher)?

Yes ☐
No ☐

A2. Please indicate the number of different courses (modules, lectures, practical courses, seminars, etc.) that you currently teach yourself or in which you participate (e.g. as a guest teacher) that intentionally address "communicating with patients" as a topic.

Number of courses:

A3. Are these different courses (modules, lectures, practical courses, seminars, etc.) that you teach yourself or in which you participate (e.g. as a guest teacher) required or elective courses?

	Required	Elective
Course 1	☐	☐
Course 2	☐	☐
Course 3	☐	☐
Course 4	☐	☐
Course 5	☐	☐
Course 6	☐	☐
Course 7	☐	☐
Course 8	☐	☐
Course 9	☐	☐
Course 10	☐	☐

A4. How many students attend your course(s) (modules, lectures, practical courses, seminars, etc.) that you teach yourself or in which you participate (e.g. as a guest teacher) that cover "communication with patients"? Please indicate the number for each course.
Course 1:

Please list numerical digits 1-499.

Attachment 1 to: Exenberger S, Kumnig M, Huber A, Prodinger WM, Siller H, Medicus E, Brenner E, Schüßler G, Höfer S. Communicative and social competence in the medical curriculum of the Medical University of Innsbruck: learning objectives, content, and teaching methods. GMS J Med Educ. 2021;38(3):Doc59. DOI: 10.3205/zma001455

A5. How many students attend your course(s) (modules, lectures, practical courses, seminars, etc.) that you teach yourself or in which you participate (e.g. as a guest teacher) that cover “communication with patients”? Please indicate the number for each course. Course 2:

Please list numerical digits 1-499.

A6. How many students attend your course(s) (modules, lectures, practical courses, seminars, etc.) that you teach yourself or in which you participate (e.g. as a guest teacher) that cover “communication with patients”? Please indicate the number for each course. Course 3:

Please list numerical digits 1-499.

A7. How many students attend your course(s) (modules, lectures, practical courses, seminars, etc.) that you teach yourself or in which you participate (e.g. as a guest teacher) that cover “communication with patients”? Please indicate the number for each course. Course 4:

Please list numerical digits 1-499.

A8. How many students attend your course(s) (modules, lectures, practical courses, seminars, etc.) that you teach yourself or in which you participate (e.g. as a guest teacher) that cover “communication with patients”? Please indicate the number for each course. Course 5:

Please list numerical digits 1-499.

A9. How many students attend your course(s) (modules, lectures, practical courses, seminars, etc.) that you teach yourself or in which you participate (e.g. as a guest teacher) that cover “communication with patients”? Please indicate the number for each course. Course 6:

Please list numerical digits 1-499.

A10. How many students attend your course(s) (modules, lectures, practical courses, seminars, etc.) that you teach yourself or in which you participate (e.g. as a guest teacher) that cover “communication with patients”? Please indicate the number for each course. Course 7:

Please list numerical digits 1-499.

A11. How many students attend your course(s) (modules, lectures, practical courses, seminars, etc.) that you teach yourself or in which you participate (e.g. as a guest teacher) which that cover “communication with patients”? Please indicate the number for each course. Course 8:

Please list numerical digits 1-499.

A12. How many students attend your course(s) (modules, lectures, practical courses, seminars, etc.) that you teach yourself or in which you participate (e.g. as a guest teacher) that cover “communication with patients”? Please indicate the number for each course. Course 9:

Please list numerical digits 1-499.

A13. How many students attend your course(s) (modules, lectures, practical courses, seminars, etc.) that you teach yourself or in which you participate (e.g. as a guest teacher) which that cover “communication with patients”? Please indicate the number for each course. Course 10:

Please list numerical digits 1-499.

A14. What learning content do you teach regarding the topic of "communicating with patients"? Please list these as keywords.

A15. Which teaching methods do you use to teach students skills for "communicating with patients"?

- Lecture ☐
- Patient presentations ☐
- Bedside teaching ☐
- Case vignettes ☐
- Discussion ☐
- Group work ☐
- Simulated patients ☐
- Role-play ☐
- Video analysis of interviews ☐
- Other ☐

Other:

A16. Which assessment formats are used to measure learning gains in the course(s) (modules, lectures, practical courses, seminars, etc.) that you teach yourself or in which you participate (e.g. as a guest teacher)?

- Written test ☐
- Oral exam ☐
- Combination of written test and oral exam ☐
- Practical assessment (e.g. OSCE) ☐
- Other ☐

Other:

A17. In your opinion, how important is teaching "communication with patients" at the Medical University of Innsbruck?

- Unimportant ☐
- Not so important ☐
- Important ☐
- Very important ☐

A18. I believe teaching competence in "communicating with patients" in medical education is:

- Unimportant ☐
- Not so important ☐
- Important ☐
- Very important ☐

A19. Have you completed special training on how to teach communication with patients?

- Yes ☐
- No ☐

A20. What type of special training to teach communication with patients have you completed?

A21. How well are you informed about the required courses, which according to the curriculum, teach communicative competence with patients?

- Extremely well ☐
- Very well ☐
- Not very well ☐

Not at all well ☐

A22. How well are you informed about elective courses, which according to the curriculum, teach communicative competence with patients?

Extremely well ☐
Very well ☐
Not very well ☐
Not at all well ☐

A23. Which routine communication skills should medical students have once they finish their education? Please list two examples:

A24. Are you interested in networking, sharing experiences, or in the development of standards on communicative competence for medical students at the Medical University of Innsbruck?

Yes ☐
No ☐

Thank you for participating!

Dr. Silvia Exenberger, Dr. Alexandra Huber, Dr. Stefan Höfer, Dr. Gerhard Schüßler, Dr. Wolfgang Prodinger, Dr. Peter Loidl

If you are interested in networking, sharing experiences, or in the development of standards on communicative competence for medical students at the Medical University of Innsbruck, please include your email address on the next page. *This data will be saved separately from the survey data.*

Thank you!
